# Contrasting Realities in Injury Management: Strategies Employed by Performance Nutritionists and Dietitians in Ireland (Part A)

**DOI:** 10.1016/j.cdnut.2025.107507

**Published:** 2025-07-24

**Authors:** Emma Finnegan, Ed Daly, Lisa Ryan

**Affiliations:** Department of Sport, Exercise and Nutrition, Atlantic Technological University, Galway, Ireland

**Keywords:** injury 1, sport 2, nutrition 3, recovery 4, athletes 5, dietary 6, supplement 7, assessment 8, tissue healing 9

## Abstract

**Background:**

Nutrition underpins athletic performance, enhancing training, reducing injury risk, and accelerating recovery. In the event of an injury, performance dietitians (PDs) and nutritionists’ (PNs) play a vital role by tailoring nutritional strategies to support tissue repair, optimize athlete’s recoveries, and return to play.

**Objectives:**

This study explored nutritional strategies recommended and employed by Irish PDs and PNs to assess, manage, and support athletes during the initial stages of sports-related injuries.

**Methods:**

Seventeen PDs and PNs across Ireland participated in semistructured interviews. All interviews were transcribed verbatim, and an inductive qualitative content analysis was applied to identify initial codes and extract relevant insights from data.

**Results:**

PDs and PNs in Ireland work across various sports and competitive levels. Their capacity to support injured athletes is influenced by contextual factors, particularly the quality of communication and interdisciplinary collaboration within their sporting environments. Participants reported variability in their initial injury assessment and nutrition management practices across sports, organizations, and genders. These inconsistencies influence the implementation of nutrition strategies and impact athletes’ subsequent recovery outcomes. In particular, limited or nonspecific nutritional support was linked slower or less effective recovery.

**Conclusions:**

The diversity in nutritional strategies employed by Irish PDs and PNs during the initial stages of injury management highlights the need for standardized, sport- and context-specific protocols to optimize recovery outcomes. Implementing evidence-based guidelines, consistency using structured assessment tools, and enhancing multidisciplinary collaboration may reduce inconsistencies and improve support for injured athletes, particularly in resource-constrained settings. Practitioner-informed strategies such as injury-specific checklists, routine assessments, coordinated care with medical teams, and targeted nutrition education, may enhance athlete buy-in and promote more effective, recovery focused interventions.

## Introduction

Globally, it is reported that between 3 and 5 million sports-related injuries occur annually, with >72% sustained during competitive events [[1], [2], [3]]. Sports injuries present as a loss of bodily function or structure caused by participation in sport, as identified through clinical examination [4,5]. Their incidence, prevalence, and type vary substantially across countries, sport type, levels, activity demands, funding, and popularity, and athlete-specific characteristics, including BMI (in kg/m^2^), gender, and age group [1,2,4,6]. Among elite and nonelite athletes, sports injuries are attributed to factors such as increased training loads, dense competition schedules, performance pressures, sport-specific physical movements, in addition to sporting culture and athlete behaviors [1,7,8]. Consequently, varied strategies are required to manage these demands, reduce injury risk, and support athletes throughout coping, recovery, and returning to play (RTP) processes.

Ireland is home to Gaelic football, hurling (male), and camogie (female) [9], as well as popular international sports, such as soccer, rugby, hockey, and cricket. Gaelic sports are commonly characterized by an amateur status and professional ethos [10]. Gaelic athletes perform at a professional intensity and standard while competing in community-based club and intercounty levels within their counties [10]. Like many field-based competitive team sports, physical contact is common and most often unavoidable, exposing athletes to increased injury risks [11]. Gaelic athletes do not have a professional status in Ireland and therefore balance their playing commitments with full-time study or employment [10]. This dual burden increases their injury risk due to cumulative physical and psychological stress, limited time for optimal nutritional intake, recovery, and sleep, and a higher likelihood of overtraining or inadequate recovery [12,13].

Current data on male Gaelic footballers identify that over 70.0%–95.0% of injuries affect the lower limbs, particularly the quadriceps, hamstrings, ankle and knee tissues [14]. Among these, hamstring tears and anterior cruciate ligament (ACL) strains are most commonly reported during match play (55.9/1000 h) compared with training (4.6/1000 h) events [10,14]. Similarly, the majority of injuries among ladies' Gaelic footballers (LGF) affect lower limbs, accounting for ∼ 67.1% of reported injury incidences [15]. As with male Gaelic footballers, these injuries predominantly affect the thigh (hamstring, quadriceps), knee (ACL), and ankle. However, ACL injuries pose the greatest injury burden for females, resulting in an average of 106.5 d of absence per 1000 h of play [8,15].

Similarly, injury rates are high among stick sports athletes in Ireland. Male hurlers experience between 61.8 and 102.5 injuries per 1000 h of competition, compared with 2.9–5.3 during training [16]. Female camogie athletes sustain an average of 26.4 injuries per 1000 h of competition and 4.2 per 1000 h during training [17]. Sprinting, landing, change of direction, and kicking movements are commonly associated with these lower limb injury events [[15], [16], [17]]. Additionally, upper body impacts to the hands, fingers, thumbs, and head from collisions or being struck by the hurley often result in fractures, concussions, and eye injuries [15,16].

Furthermore, this trend is observed in Irish male and female soccer athletes, who are predominantly affected by lower limb injuries, with over half resulting from noncontact mechanisms impacting the upper thigh. Common injuries include strains to muscle and ligament tissues, such as the hamstrings, adductors, and quadriceps, as well as cartilage damage to the knee, and the ankle. Similar to Gaelic sports, ACL injuries also represent the highest burden [18,19].

Injury patterns for Irish rugby athletes follow a similar trend, with most affecting the lower extremities and head. The Irish Rugby Injury Surveillance 2023–2024 report identified concussion (12% of injuries, averaging 30 d’ absence), followed by ankle and knee ligament sprains (9%, averaging 36 d), as the most burdensome injuries among male athletes [20,21]. For female athletes, 11% of injuries were ligament sprains to the knees and ankles, resulting in an average absence of 30 d for ankles and 162 d for knees. Concussion (9%) led to an average of 28 d’ absence. These injuries significantly affected player recovery and team availability [20,21], with injury rates also influenced by playing positions. Forwards (front, second, and back rows) experienced a higher incidence of head and shoulder injuries, likely due to the physical and contact-heavy nature of their roles. In contrast, backs (halves, inside and outside backs) were more prone to ankle injuries, often from noncontact movements such as rapid changes in direction [22].

Field hockey, an amateur sport in Ireland, reports injury rates ranging from 7.8 – 11.8 per 1000 h [23]. Common injuries include muscle strains, pain, and contusions, predominantly affecting players’ lower limbs, the hamstrings, knee, hip, and groin areas. Noncontact mechanisms account for 66.9% of injuries, with 16.1% classified as recurrent [17]. Injuries resulting from stick or ball contact and player-to-player collisions also contribute significantly to the overall injury burden [23].

Injuries are an inevitable aspect of sport; therefore, mitigation strategies such as prevention programs to enhance tissue strength and conditioning [8] along with improved dietary practices and behaviors [[24], [25], [26]], are essential to reducing injury, illness, and long-term health complications [18,27]. Understanding injury incidence, particularly the frequency and types of common injuries, helps inform targeted nutrition strategies that support both injury prevention and the optimization of rehabilitation and RTP protocols. These strategies often prioritize high-burden injuries, especially those affecting lower limb muscles and ligaments [1,18,25].

Injuries cause complex physiological, metabolic, and psychological disturbances as the body strives to restore balance and return to its preinjury state [27]. Nutrition for injury management is an emerging field within sports science, with promising evidence for enhancing tissue healing and supporting recovery during injury periods [1,3,25,[27], [28], [29], [30]]. Proper nutrition can reduce risk of surgical complications, prevent muscle loss, support tissue healing, optimize RTP, and reduce overall injury burden [3,28]. Physiological mechanisms activated within the first 72 h after injury increase energy demands to support cellular repair and initiate tissue repair processes [3,4,27]. However, an athlete’s nutritional status at the time of injury influences both their risk of injury and recovery, with outcomes shaped by injury type, severity, treatment, and individual factors [3,27]. Adequate protein intake stimulates muscle protein synthesis and limits atrophy caused by disuse or immobilization. As the human body has no reserve of protein, insufficient intake leads to the breakdown of functional tissue [3,27]. Injury recovery requires enough energy and nutrients to rebuild tissue. Carbohydrates (CHO) replenish glycogen stores (muscle and liver), maintain energy, enhance immune function, and reduce muscle protein breakdown. CHO-rich diets may spare muscle protein more effectively than high-fat diets during injury-induced catabolic states [3,27]. Additionally, ω-3 fatty acids help modulate inflammation and support cardiovascular and brain health [31]. Micronutrients such as vitamin D_3_ and calcium contribute to bone density, tissue repair, immune regulation and muscle function, making them essential for recovery [3].

In Ireland, performance dietitians (PDs) and nutritionists (PNs) manage athletes’ nutrition by applying evidence-based dietary and supplementation strategies to enhance tissue healing post injury [28,32]. Tissue healing occurs across 3 overlapping stages (S) inflammation (S1), repair and proliferation (S2), and rehabilitation and remodeling (S3) [4,27]. S1 is an acute, innate, energy-intensive inflammatory response, characterized by increased blood flow and cytokine activity at the injury site to break down and remove damaged tissue [4]. S2 and S3 are primarily anabolic. S2 begins tissue rebuilding through collagen formation and muscle regeneration, whereas S3 involves restructuring and strengthening of repaired tissues. Prolonged inflammation during S2 and S3 is not conducive to recovery [3]. Each stage requires specific macronutrients (CHOs, fats, and proteins), and micronutrients (vitamins and minerals) to support healing and recovery, especially during the anabolic stages (S2 and S3) [3,27,30].

However, PDs and PNs require a clear understanding of injury incidence within their athlete cohorts to effectively manage nutritional strategies during recovery [25,28]. Currently, little is known about the nutrition strategies utilized by Irish PDs and PNs to facilitate athletes’ injury recovery or the potential gaps in their practices.

Therefore, this study aimed to explore the nutritional strategies recommended and employed by Irish PDs and PNs to assess, manage, and support athletes during the initial stages of sports-related injuries. It will also examine how practitioners adapt their approaches based on injury type, sport resourcing, athlete context, and multidisciplinary collaboration.

## Methods

An inductive qualitative content analysis was employed to examine interview data from PDs and PNs to extract coherent meanings and identify nutritional strategies used in practice to manage and support injured athletes’ recovery and RTP [[33], [34], [35]]. The Consolidated Criteria for Reporting Qualitative Research guidelines were adhered to throughout the interview design, conduct, and reporting processes [36].

### Researcher reflexivity

To ensure reflexivity and minimize bias, the lead researcher (EF) engaged in bracketing to reflecting on professional backgrounds in sport and nutrition consultancy, across community, and applied sport settings, thereby reducing the influence of preconceptions prior to data collection [37]. Reflexivity acknowledges that EF’s positionality and experiences influence the study focus and interpretation, while actively managing potential biases to ensure an objective exploration of how nutrition practitioners support injured athletes across Ireland [38]. A reflexive journal recorded assumptions, emotional responses, and evolving interpretations before, during, and after interviews [39]. Analytic memoing recorded decisions during data analysis [33]. Peer discussions with coauthors (LR and ED) throughout the analysis process, including reviews of reflexive notes, enhanced transparency, mitigated individual bias, and strengthened the trustworthiness of findings.

### Sampling and eligibility criteria

Purposeful sampling was implemented [40,41] to ensure diversity in practitioner responses, across experience levels, sport settings, and athlete demographics. This approach aimed to capture a comprehensive perspective on injury management and nutrition strategies within the Irish sporting context, by including practitioners who had experience working in various sports (e.g., rugby, Gaelic football), across elite, subelite, and/or amateur levels.

Participants were identified through professional registrations (the Sport and Exercise Nutrition Register), national sporting bodies, such as the Irish Rugby Football Union (IRFU), the Gaelic Athletic Association (GAA), Sport Ireland, and sports nutrition networks.

Eligible participants were required to be registered to practice in or have practiced in Ireland within the last 10 y (at the time of recruitment) to obtain a comprehensive Irish perspective on performance and sports dietetics and nutrition. They must also have had experience working with adults and youths (≥16 y of age), across both team and/or individual sports, and at professional/elite, subelite, and/or amateur levels.

Eligible candidates were invited to participate in 1–1 interviews online via email by EF, who was available to address any questions or queries regarding the interviewing process and data collection. Sample size was determined based on data saturation, defined as the stage at which no new themes or codes emerged, indicating information redundancy [42]. In this study, the sampling process began in March 2022, with data saturation being achieved after 17 interviews in August 2022. Although participants were recruited from a range of Irish sporting contexts, there was a greater representation from practitioners working within professional and Gaelic sports. This reflects how elite sport is structured in Ireland but may limit the transferability of findings to less structured or resourced sporting environments.

Semistructured interviews were conducted, allowing questions to accommodate PDs/PNs with differing backgrounds and levels of experience with athletes. Before the study commenced, the interview guide was piloted with 2 PNs to evaluate question appropriateness and ensure logical flow of the interview script. The interview format was designed to gather information on participants’ background, education and career to date, the types of athletes they worked with (age group, gender, sporting level, etc.); their sport(s); and the settings in which they were involved (e.g., professional, amateur, community) as well as their experience with injury management. Participants included both females and males working with athletes and/or teams classified as professional/elite, semiprofessional/subelite, or amateur. At the time of the interview, all athletes supported by PDs/PNs were based in the Republic of Ireland or Northern Ireland.

### Ethics and procedure

Ethical approval was granted by the Atlantic Technological University Research Ethics Sub-Committee of Academic Council (ATU-RSC_AC_25/11/2021). Participants (*n* = 17) provided informed consent online via Microsoft Forms [43] before scheduling interviews. Prior to each interview, EF held informal discussions to clarify the study objectives, procedures, and confidentiality measures. Interviews were then conducted by EF using the online video conferencing platform Microsoft Teams [43].

As a final confirmation before the interview commenced, participants provided verbal consent which was audio recorded. Interview durations ranged from 36 – 54 min (mean = 47.77 ± 9.9 min). At the end of the interview, participants were reminded that all information would be treated confidentially and fully anonymized for research purposes.

### Data collection

Interview questions gathered evidence of PDs’/PNs’ personal experiences with injuries within the athlete cohorts they supported in a performance nutrition capacity. Participants discussed their awareness of injury events, including injury classifications and types, and described their approaches and roles in injury management.

### Data analysis

After data collection, the lead researcher (EF) transcribed each interview verbatim, listening to the audio recordings multiple times for verification. Transcripts and recordings. were examined for accuracy (e.g., mispronunciations) and reread repeatedly to enhance trustworthiness [33,34]. A second author (LR), blinded to participant identities, also reviewed the transcripts to ensure accuracy and further support the credibility of the data. Initial patterns and trends identified in the transcripts were discussed among the research team (EF, LR, and ED), leading to the development of a preliminary data categorizing framework. This framework was refined iteratively throughout data collection to ensure data coherence and inclusivity of emerging patterns.

An inductive qualitative approach was primarily used, incorporating elements of reflexive content analysis to ensure the analysis remained grounded in participants’ language, experiences, and meaning, rather than being shaped by a predetermined or rigid theoretical framework [39]. This approach was selected to explore how Irish PDs and PNs support injury management in their practices, and to generate rich, practice-based insights and inform theory-building rooted in applied contexts.

**FIGURE 1:**
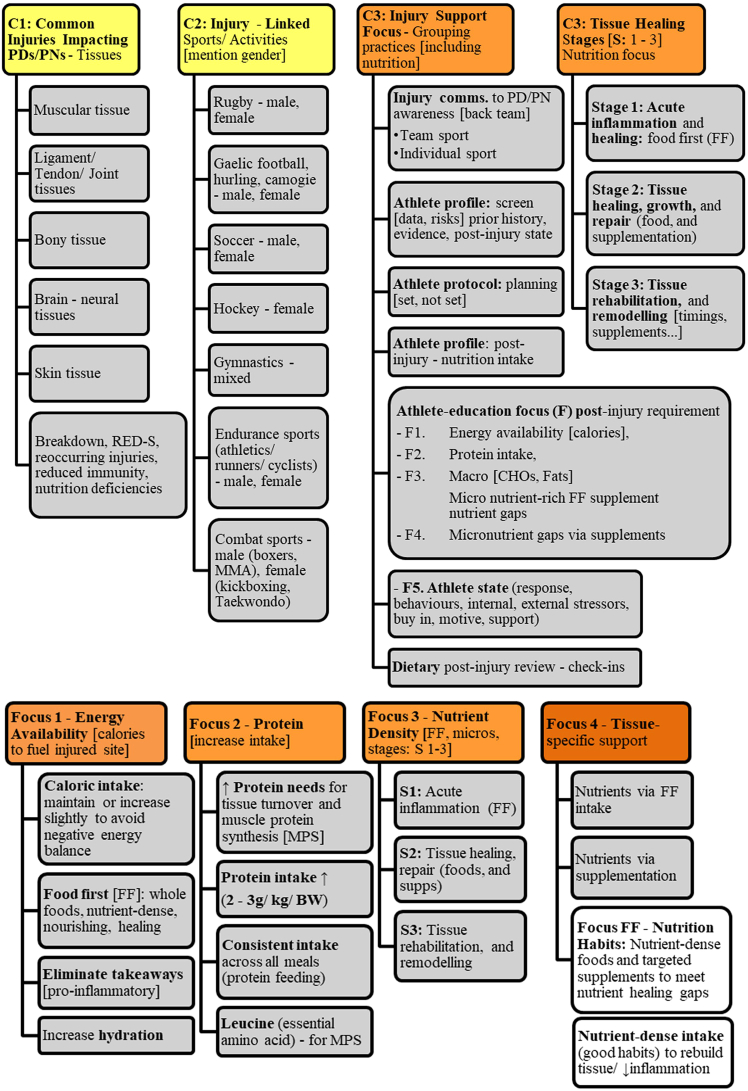
Categories identified from interview data organizing and categorizing injury management practices. BW, body weight; C, category; CHO, carbohydrates; Comms., communications; F, mentioned focus; FF, food first; MPS, muscle protein synthesis; N, nutrition; PD/PN, performance dietitian/nutritionist; RED-S, relative energy deficiency in sport; S, stages of tissue healing (S1, S2, S3); ↑, increase or surplus; ↓, decrease or reduce.

Data were organized and categorized manually by the research team using Microsoft word and Excel [43], without the use of qualitative analysis software. The process began with open coding, followed by data categorization into broader themes based on recurring patterns. Open coding, categorizing, and abstracting were initially used, followed by refinement of categories into more detailed codes and subcategories. These were linked to create a map of conclusions based on abstracted meaning units aligned with the study’s purpose (Figure 1). The hierarchical framework developed through inductive coding was further informed by physiological models of tissue healing, ensuring the categorization reflects both participants’ experiential data and relevant biological processes. Although *phases* is the term commonly used in scientific literature, participants referred to these processes as *stages* [4]. To maintain consistency and reflect participants' language, this terminology is used throughout the results and discussion. Throughout the analysis, the research team engaged in discussions to ensure consistency and rigor in interpreting the data.

## Results

In line with the study aims to explore the nutritional strategies employed by PDs and PNs initially after an injury, and the contextual factors shaping these strategies, the following findings emerged through inductive content analysis.

### Overview of findings

Content analysis of interview data identified common steps PDs/PNs employed to manage and prevent injuries, influenced by factors such as the athlete, their environment, the type of injury, and its healing requirements. These factors led to differing levels of support and nutritional strategies utilized in practice by PDs/PNs. These steps were categorized into an initial screening and injury management framework to help streamline nutrition protocols into clear, actionable steps relevant to practice. This approach provides a rationale for the best practices followed by PDs/PNs in Ireland while identifying gaps in injury management. Therefore, PDs/PNs will be supported with the best practices relevant to their environment and nutritional practical guidelines to implement and support athletes in achieving full health, recovery, and RTP.

### Participant characteristics

Participants were Irish PDs and PNs working across professional/elite, semiprofessional/subelite, and amateur levels in the Republic of Ireland and Northern Ireland. All participants held a minimum of a Master of Science degree in sports nutrition and exercise and/or a Doctor of Philosophy (PhD) (Table 1 and Supplemental Table 1).

**TABLE 1:** Participant profile: performance dietitians and nutritionists background, education, injury history, and athlete environment.

Participant characteristic	Total sample (*n* = 17)	Percentage (%)
**Gender**	***n***	**%**
Male	8	47.1
Female	9	52.9
SENr registered at time of interview	17	100.0
**Undergraduate degree**		
BSc dietetics and nutrition sciences	2	11.8
BSc human nutrition sciences	3	17.6
BSc nutrition and sports sciences	1	5.9
BSc health, sport, and exercise sciences	6	35.3
BA education with science	2	11.8
Other (BA, B Com, BBS nonscience degrees)	3	17.6
**Postgraduate degree** (MSc/ PhD)		
MSc in sports nutrition	4	23.5
Dip sports nutrition	3	17.6
MSc sports and exercise nutrition	9	52.9
MSc in human and/or nutrition	2	11.8
MRes in sport and exercise nutrition	1	5.9
Doctor of philosophy (PhD) 1	5	29.4
**Injury experience**		
With their athletes	17	100.0
Personal (as an athlete)	6	35.3
**Employment contract/role(s)**		
Part-time PN role	8	47.1
Full-time PN role	6	35.3
Full-time self-employed PN	3	17.6
**Athlete environment at time of interview**		
**Team-based support**	14	82.4
• Part-time—team (*multiple*)^2^	8	47.1
• Full-time—team sport setting^3^	6	35.6
**Nonteam-based support**	10	58.8
• Part-time—individual (including teams)^2^	7	41.2
• Full-time—individual—self-employed	3	17.6
**Sport/athlete type**		
- Men’s Gaelic football	14	82.4
- Ladies Gaelic football	4	23.5
- Hurling	7	41.2
- Camogie	2	11.8
- Men’s rugby	10	58.8
- Women’s rugby	5	29.4
- Men’s soccer	4	23.5
- Women’s soccer	3	17.6
Hockey	3	17.6
Cricket	1	5.9
Endurance sport (e.g., running, cycling, triathlon)	8	47.1
Combat sports (e.g., boxing, MMA, judo)	6	35.3
Gymnastics	1	5.9
Strength and power (e.g., weightlifting, powerlifting)	3	17.6

Abbreviations: BA, bachelor of arts; BBS, bachelor of business studies; BCom, bachelor of commerce; BSc, bachelor of science; Dip, diploma; MMA, mixed martial arts; MRes, research masters of sciences; MSc, master of science; *N*, participant full sample, PD/PN, performance dietitian/nutritionist (participants); PhD, doctor of philosophy; SENr, sport and exercise nutrition register; ToI, time of interview.

1 Field/specialism areas include energy, sports and exercise nutrition, bone health, exercise biochemistry, and bioactive compounds for health and performance.

2 Part-time contracts/multiple team roles refer to PDs/PNs reporting part-time involvement with multiple (1+) sports teams (PN1, PN3, PN4, PN7, PN11, PN12, PN15, PN17).

3 Full-time team roles were held with Irish sporting organizations [Sport Ireland, the Gaelic Athletic Association (GAA), and the Irish Rugby Football Union (IRFU)] (PN2, PN5, PN8, PN9, PN13, PN14).

PDs and PNs supported athletes in both team-based and individual sport contexts. Team sports included Gaelic football (men’s, 82.4%, *n* = 14; ladies, 23.5%, *n* = 4), hurling (41.2%, *n* = 7), and camogie (11.8%, *n* = 2), across intercounty, county, and club levels. Rugby union was represented by both men’s (58.8%, *n* = 10) and women’s (29.4%, *n* = 5) teams, for amateur, club, and professional or elite levels. Soccer included male (23.5%, *n* = 4) and female (17.6%, *n* = 3) athletes competing in the Premier League, League of Ireland, and club settings. Other team sports included women’s hockey (provincial and club level) and professional cricket. In individual sport contexts, PDs/PNs worked with athletes in combat sports (35.3%, *n* = 6) including boxing, mixed martial arts, kickboxing, and judo across both amateur and elite levels. They also supported elite gymnasts and endurance athletes, including those involved in running and cycling (47.1%, *n* = 8), across elite, ex-elite, and amateur levels. Additionally, participants worked with athletes in rowing and multisport disciplines such as triathlon, Olympic weightlifting, and powerlifting. At the time of interview, 82.4% of participants (*n* = 14) worked in team-based environments. Of these, 8 (47.1%) held part-time roles across multiple teams, and 6 (35.6%) were employed full-time within national governing bodies such as the GAA, IRFU, and Sport Ireland. Nonteam-based roles (58.8%, *n* = 10) included part-time consultancy and full-time self-employed support for individual athletes, often in endurance or combat sports.

**FIGURE 2:**
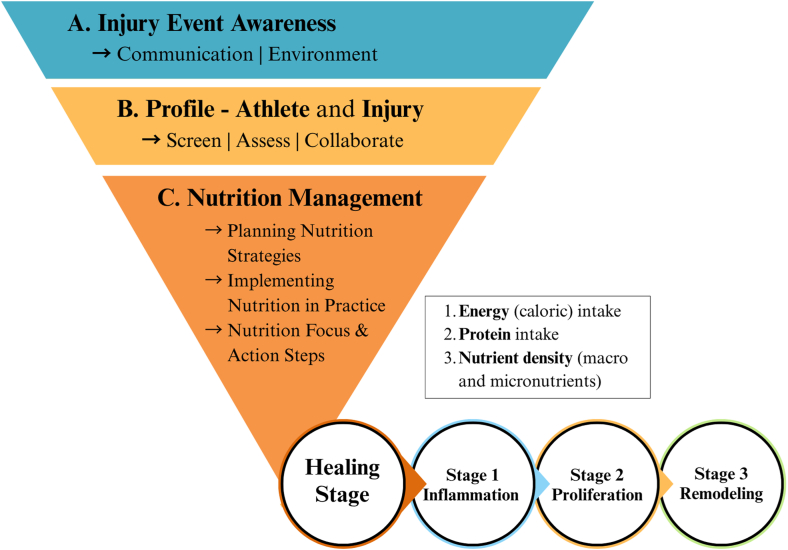
Organizing and categorizing injury management data as described by performance dietitians and nutritionists. S, stages of tissue healing (S1: stage 1, S2: stage 2, S3: stage 3).

This article focuses on initial injury management, including actions taken during the 3 healing stages (S1, S2, and S3) (Figure 2). Each category is summarized with references to relevant figures that illustrate actions, strategies, and participant quotations to highlight key narratives and practical applications.

### Injury types and context

This results section categorizes (C) the strategies implemented by PDs/PNs in Ireland in order of cited importance, frequency of recommendation, and practical application in managing and preventing sports-related injuries (Figure 1: C1, C2). Figure 1 presents the main injury types (C1), the corresponding athlete populations and sports (C2), and the tissue healing stages with associated nutrition strategies (C3, C4).

Common injuries included muscle, ligament, tendon, bone, and brain injuries, along with less visible issues such as relative energy deficiency in sport (RED-S), recurring injuries, compromised immunity, and nutrient deficiencies. PDs/PNs supported elite, subelite, and amateur athletes across a diverse range of team and individual sports, including rugby, Gaelic football, soccer, hurling, camogie, hockey, gymnastics, endurance events, and combat sports. A key challenge identified was that PDs/PNs often became aware of injuries only after athletes had noticeably withdrawn from training, matches, or competition, limiting opportunities for early-stage nutritional intervention.

Participants described a shared, step-by-step approach to managing these injuries, focusing on the specifics of each injury scenario and athlete context. A consistent hierarchy of nutritional priorities emerged, with emphasis on energy availability, adequate protein intake, and nutrient-dense foods. Several participants detailed strategies aligned with tissue healing stages (Figure 1: C3, C4), from acute healing (S1) through to rehabilitation and tissue remodeling (S3) [4,27].

Despite these efforts, PDs and PNs reported several barriers to effective injury management. These included both avoidable and unavoidable organizational and interpersonal stressors, such as limited access to multidisciplinary support teams, funding constraints, athlete burnout, under-fueling, and inconsistent engagement from the athletes and/or coaches. Communication breakdowns frequently delayed the delivery of timely nutritional support. Subtle “*niggles*” and recurrent injuries were often early indicators of underlying nutritional or recovery issues, but these were not always recognized or reported promptly. Communication around injury status significantly influenced PDs’/PNs’ awareness, shaping the timing and nature of nutritional interventions provided.

**FIGURE 3:**
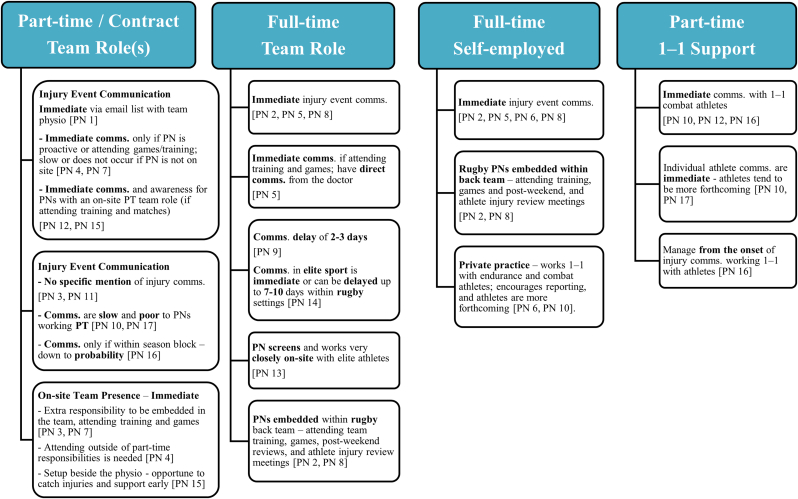
Degree of injury event communication mentioned by performance dietitians and nutritionists, categorized by contract type. 1-1: individual athlete support (nonteam environment); comms.: communications; PD/PN, performance dietitian/nutritionist; PT, part time.

As illustrated in Figure 3, communication practices varied among practitioners. Ideally, immediate injury notification, access to relevant athlete health data, and the use of screening tools would facilitate earlier and more targeted nutritional strategies to support tissue healing. However, this level of integration was rarely achieved in practice among the PDs and PNs interviewed (*n* = 17).

### Initial injury communication

Effective communication was identified as essential for PDs’/PNs’ injury awareness and timely nutritional intervention (*n* = 17), illustrated in Figure 3. Although 70.6% (*n* = 12) of participants reported receiving immediate injury communication, their experiences varied considerably. Some PDs/PNs worked beyond their contracted team h, attending matches or training sessions to be informed about potential injuries (PN 4, 7).

Within professional rugby environments, PN 9 described typical delays of 2–3 d injury communication, PN 14 reported delays of 7–10 d, and PN 17 experienced irregular updates due to part-time involvement and contractual limitations. In contrast, PN 2 and PN 8 received immediate communication as they were full-time onsite and participated in regular post-weekend athlete back-team review meetings, enabling early nutritional intervention. PN 14 also reported immediately communicating with their elite athletes and teams (in gymnastics, hockey, athletics, cycling). PN 15 shared that they would intentionally position themselves beside the team physio at matches to catch early injury communications.

In contrast, PN 10 experienced slower or no communication with Gaelic team athletes, unlike the prompt injury communication reported when working with their individual combat athletes. Similarly, PNs 6, 16, and 17 too indicated immediate communication with individual athletes. PN 5, working with Gaelic athletes, reported receiving immediate injury communications when onsite, during training and matches, as well as messaging updates on athlete status, due to a strong, open relationship with team doctors and physiotherapists.

**FIGURE 4:**
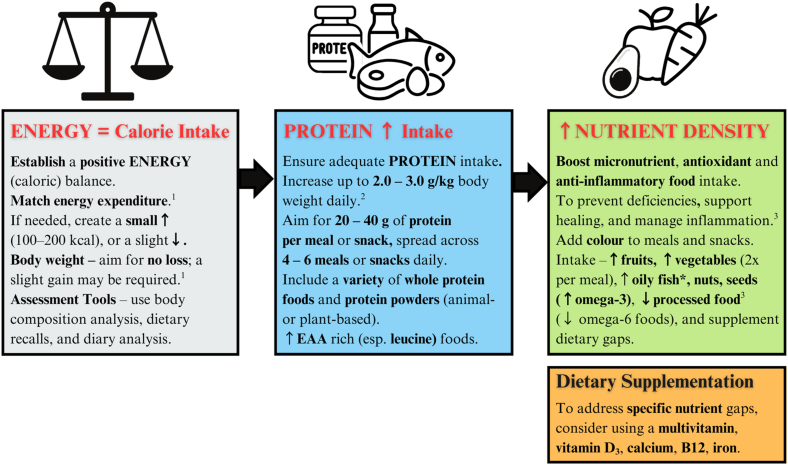
Primary initial nutrition management focuses post injury, as mentioned most by performance dietitians and nutritionists. Detailed nutrition management areas include energy, protein, and nutrient-dense foods. EAA, essential amino acids; Esp., especially; FFM, fat free mass; PD/PN, performance dietitian/nutritionist; ↑, increase; ↓, decrease; BMR, basal metabolic rate. ^1^Energy: BMR × stress factor × activity level. Avoid chronic low energy availability (<30 kcal/kg FFM/d). Target energy for recovery is ∼45 kcal/kg FFM/d [27]. ^2^Protein: should be ≥1.2–1.6 g/kg/d, with an upper target of 2.0–3.0 g/kg/d [1,3,27,30,44,45]. ^3^Oily fish provide anti-inflammatory benefits (↑ ω-3), while processed foods are proinflammatory due to high ω-6 intake. Maintaining a low ω-3 to ω-6 ratio is important to reduce inflammation and support recovery post injury [3,27].

Four practitioners (PNs 1, 3, 11, and 13) did not specify injury communication timeframes (23.5%, *n* = 4/17). PNs 3 and 11 attended training and matches part-time, and PN 13 was full-time onsite with their elite athletes (FIGURE 3, FIGURE 4) [1,3,27,30,44,45]. PN 1 mentioned relying on mailing lists to be informed about injury incidents. However, PN 1 indicated that a developing relationship with the team physiotherapist helped them be “*privy to any new injuries that may have occurred*” (PN 1); they did not mention receiving immediate injury reports when asked directly, suggesting that communication may not always be prompt.

PDs/PNs discussed that “*communication, in every sense of its form*” (PN 6) was vital when an athlete is injured or at risk, as it shapes postinjury assessments, screening, nutritional support, and management. However, effective communication requires trust and collaboration among coaches, physiotherapists, medical, and other support staff within the athlete/team ecosystem. Communication needs to be,

“*…between the different corners of the sporting industry… from physio… to the medical team on these sporting teams, and individual sports… Ehmm, to nutritionists, to their family, and their friends to get everybody… communicating… as a support team behind the athlete, I think will really help in terms of making them feel fully, fully, supported*” (PN 6).

Multidisciplinary team communication is essential for injury incident awareness and enables PDs/PNs to gain a clearer understanding of the nature of injuries better. As PN 17 stated in a professional setup, discussions around injuries involve “*very technical language*” (PN17). The dynamics between the PDs/PNs and the other support staff, including athletes, play a crucial role in communication and influence athletes’ subsequent recovery and RTP.

Delayed communication, such as learning about an injury a week late, leads to missed healing windows, which PDs/PNs find frustrating. One participant cited “…*I found out about an injury a week later, like, I didn’t know where this guy was? Can someone give me a shout? So, I could have a chat with him; it’s important!*” (Gaelic athlete, PN 4). In contrast, PN 17 described a more collaborative environment within their rugby setup, sharing that “*physios are quite good at saying will you touch base with this player...*”.

PN 4, working part-time with Gaelic team athletes, described feeling pressured by unrealistic expectations to “*catch everyone… and every catch injury”* while juggling multiple roles, especially when other back team members do not equally value or “*don't really believe in all the different pillars as much as their own pillar”* (PN 4). Communication ebbs and flows, and PDs/PNs have a responsibility to stay engaged with their athletes to remain aware of injuries and identify the most opportune times to intervene, as highlighted by PN 15.

“*It’s a really important point you’ve made, which is ‘when do you become aware?’ and that like it’s very easy to give out to people, ‘ohhh, I never heard about that!’ and it’s a week later… and you didn’t get to hear… we should have a better system…*

*But sometimes you (*the PN*) just got to look in the mirror and be more responsible for how well you’ve... submerge yourself in the group. That the physio feels like you can add value… or the coach, or the manager. I do think that’s kind of an important point… you can’t just expect people to turn around and tell you that someone has been injured*” (PN 15).

Poor or delayed communication, collaboration, and strained relationships negatively impact athletes’ recovery trajectories, especially the immediate (acutely) phase after injury (see PN 9 example below), or the role of PDs/PNs is recognized or valued within the team. As PN 4 stated, their ability to provide acute nutritional support during injury and rehabilitation is at risk of being compromised. Such situations occur, for example, “*with the S&C coach and the nutritionist”*, where a “*tension”* exists that should not be present “*between certain areas of the backroom team*” (PN 4). Instead, PDs/PNs emphasized that the backroom team should “*work collectively as a team*” or operating like a “*well-oiled machine*” (PN 10), underpinned by mutual trust, shared expertise, and a unified goal aligned with their athlete’s best interests. However, participants also reported that limited understanding and education about the PD/PN role often dictated the extent of their involvement.

As PN 13 explained, nutrition support is frequently deprioritized compared with other disciplines

“*The first place is the money they’ll try, and look save… ‘Ah well, we don't need the nutritionist on that trip!’ They’ll let you know maybe a week before, and that’s just not good. They'll take their physio; they’ll take their S&C... There is lesser importance on the nutritionist service as a whole… when you look at other disciplines*” (PN 13).

PN 15 mentioned that, based on their experience, being set up *“…near the physio room or in the physio room for where you might see people, you get that first point of contact*” for more informal communication, earlier intervention, reduced formality, and better insight into the athlete’s emotional state and readiness for nutritional support. As PN 15 described:

“*…if you're in a better place to have the conversation when you can tell how that individual is… conversing with the physio if it’s jovial and it’s like, ‘ahhh… I have a pain in the arse, and I’ve got a really bad dead leg… I don’t know what to do about it?’”* (PN15).

In addition to communication, athletes’ behaviors, perceptions, and “*buy-in*” around nutrition significantly influence PDs’/PNs’ ability to support RTP and recovery. Some athletes are “*delighted to hear they should be eating more*” (PN 11) to support their performance, viewing nutrition as an opportunity to optimize recovery and avoid “*delaying their recovery process*” (PN 16). Others require repeated “*reinforcing*” to eat adequately, with PDs/PNs describing the difficulty of *“trying to basically convince them or get that buy and that you still need to fuel, and you still need to eat… enough!”* (PN 8).

Athletes “*tend to value body composition more than they value performance*” (PN 10), especially when their training load drops from “*100 to 0... they are off feet… are not doing any on feet training, and it’s just gym work*” (PN 8). This shift makes it “*awkward*” for PDs/PNs to ensure athletes eat enough to support tissue repair and prevent further injury (PN 11). As PN 10 cited:

“…*undereating comes into play, and if you have an athlete that has already been undereating… they get injured and, your or… I’m trying to get them to increase their calories… They’re going to be like, what are you on about? Do you know? Like, why am I doing this?”* (PN 10).

Undereating and low energy availability were linked to athlete behaviors. Some athletes are “*not meeting their energy needs,”* which limits PDs/PNs ability to support them, “*weakening all their systems, and that’s why they’re getting injured*” (PN 11). These behaviors are often driven by athletes’ personal value systems. As PN 5 vividly shared,

“*They want to train, and they want to play, and they don’t really want to think about nutrition yet.*.. *But, in my experience over the last two and half years, that changes gradually*” (PN 5).

Buy-in also dictates PDs’/PNs’ ability to emotionally connect with athletes’ post injury. As PN 15 shared:

“*…this is my perception*; *they don't want to be told a huge amount about nutrition until they’ve got some piece of concrete information. Like anyone when they see that they are going to be 3 weeks OUT (stressed tone). That would be my time to shine… jump in and to say, ‘look, I can’t say it will be better than 3 weeks, but we might be able to make you stronger by the time you get back in 3 weeks*” (PN 15).

Athletes’ buy-in is built on understanding why they might consume “*…two, two thousand nine hundred calories on this day when I am not even really that hungry? Or I don't need that food, or I could skip this meal*” (PN 10). At injury onset, if injured athletes do not “*understand the nutritional aspect of recovering from an injury… at the time they don’t actually have the conversation because they don’t realise that they should contact you”* (PN 4). Athletes need to understand that nutrition and what they eat will and *“does help*” (PN 4).

PDs/PNs use simple and specific educational messaging to reduce food fears and help athletes understand nutrition’s role in recovery. As PN 10 explained, “*once they get the buy into that, it is easy*” with athletes questioning strategies less and beginning to “*trust you*” (PN 10). Without this trust, undereating can be an issue. Educating athletes to use nutrition as a recovery tool empowers them to take action and “*support their recovery of that injury with nutrition… so, they’re doing something about it as opposed to feeling like they’re a little bit lost or they’re relying on maybe waiting to be able to move again*” (PN 17).

Communication also builds trust and buy-in. From day 1, PN 6 encouraged athletes to contact and report immediately “*from the minute, even if it’s at the weekend to text… from there inflammation... metabolites are starting to circulate… we are starting to see it changes to their metabolism from there on with their injury*” allowing for timely acute support (PN 6).

However, in some Gaelic teams, particularly female squads, injury management beyond communication and nutrition is not always straightforward. As PN 7 observed, professionally “*the male game gets quite a lot of fast tracking when it comes to injuries,*” whereas, personally their female teammates “*are waiting weeks or months to get in to get seen by anyone… from an injury management point of view as opposed to nutrition*” (PN 7). Presenting gender-related disparities rooting in the cultural context of Gaelic sports.

### Initial injury assessment

After an injury event, the immediate topic of conversation for PDs/PNs to consider is not specifically nutrition. Instead, practitioners assess athletes’ physical and mental states through open, informal interactions before introducing nutrition into their injury recovery conversation. PDs/PNs are aware that nutrition is “*just one piece”* of the injury management *“jigsaw*” and state that strategies need to be tailored to fit athletes’ postinjury scenario, considering competitive seasons and external pressures, such as in subelite or elite sports environments. As stated by PN 13:

“*World Cup is coming up this summer, and they’ve incurred an injury before Christmas, they’re also managing schoolwork, and now they’ve incurred another injury that has put them out of training… they are going to be worried about selections…”* (PN 13).

Outside of their sport, athletes also “*have a lot going on...*” aside from injury. As PN 4 shared, intercounty Gaelic footballers have,

“…*a full-time job or full-time study… they have to deal with the physio and the doctor. The manager is probably on their case, and then they might go, you know. I am not going to contact the nutritionist. I have to be proactive… being careful even when I am being proactive that they still might not want to take that conversation”* (PN 4).

Some PDs/PNs demonstrate strong emotional intelligence, as they “*hear the human behind the voice*” when communicating with injured athletes (PN 9). Although their qualifications open doors to help athletes, their experience enables PDs/PNs to understand, form empathy, communicate, and take meaning from each human conversation (PN 9) with injured athletes. Facilitating these open discussions, likened to “*life coaching conversations that have a nutritional element,*” helps build trust and rapport (PN 9), providing PDs/PNs insight into athletes’ overall physical and psychological readiness post injury before addressing nutrition. PN 13 stressed the importance of asking athletes, “*How are you feeling?*” with regular check-ins to gauge their needs and readiness for support.

Once PDs/PNs felt their athlete was mentally prepared, some mentioned their next focus was assessing nutrition and conducting screening. PN 5 highlighted the importance of evaluating the athlete’s food and nutrient intake to support them in tailoring nutrition recommendations and strategies. PN 8 and 10 also reinforced this approach, stating their priority is to evaluate athletes’ daily nutrition and introduce manageable nutrition changes to avoid overwhelm. PN 17 shared that they would sit down, 1–1, with their injured athletes and,

“…*look at their overall nutrition… So, I hopefully would have an understanding of, you know, what that athlete’s current nutrition is if I know them and if I*’*ve worked with them... Measure and look at athletes*’ *food intake …that will come from again, like maybe a dietary analysis. You know, or you*’*re 24-hour recall… looking at that in a little bit more detail”* (PN 17)

This process involved assessing the injury severity, determining whether health screening data were available or needed, and considering if the athlete had any or had any upcoming surgeries. Many PDs/PNs shared using a general, comprehensive approach to nutrition, without a specific focus on injury, “*ticking all the boxes, seeing what they are doing and not doing*” (PN 1). However, those who frequently managed injuries adopted more targeted approaches, focusing on specific tissues and stages of healing and aligning their nutrition strategies with recovery and rehabilitation phases [2,3,27,30]. Not used by all PDs/PNs, protocols were applied in a step-by-step manner, tailored to the type of injury and its tissue healing stage, supporting athletes such as rugby players (PN 2, 8, 9, 17), Gaelic players (PN 5, 17), and endurance runners (PN 6).

“…*there are no set resources as such, but it’s just my own step-by-step process which has been developed and moulded from the step-by-step process”* used in their current and past team setups (PN 17).

### Injury screening data and history

These screening assessments provide a clearer picture of athlete’s current health and nutritional status, highlighting areas that need focus to facilitate healing and recovery. However, consistent use is essential to build meaningful longitudinal data on athletes’ health and injury history. Participants who used these assessments had identified athletes with deficiencies in calories, macronutrients (CHOs, fats, and proteins) and key micronutrients such as calcium, iron, both before and after injury events.

Only 5 PDs/PNs [29.4%, *n* = 5/17; (PN 5, 9, 10, 13, and 14)] reported having sufficient access to resources that enabled them to perform yearly screening. These practitioners used objective assessments to gather “*evidence*” linking athletes’ injuries to nutrition and training history, helping to uncover contributing factors. Conversely, those without access recognized these tools and wanted to use them but were hindered by resource constraints. As PN 14 stated, in resource-limited settings, “*you’ve got to play what is in front of you*” and adapt to the athletes’ context to provide meaningful, practical support. In contrast, PDs/PNs working within elite, well-funded, and supported team environments reported conducting comprehensive screening at the start of each season and post injury.

PDs/PNs cited using or wanting to use objective assessments to evaluate athletes’ physiological status post injury. This included using blood biochemistry, bone density scans [dual-energy X-ray absorptiometry (DXA)], anthropometric measurements [such as skinfolds following the International Society for the Advancement of Kinanthropometry (ISAK) standards, and body weight], tools such as the low energy availability (LEA) questionnaire, and dietary analysis. DXA scans were used to assess athletes’ bone health and density, especially in cases of recurrent injury. Blood biochemistry was cited to measure glucose, ferritin, cholesterol, vitamin D and ω-3 levels, white blood cell counts, and inflammatory markers. These standardized blood panels are conducted routinely for athletes within Sport Ireland (PN 3, 13, 14). However, the IRFU enforces a “*no needles policy*,” so PDs/PNs must request blood assessments through the team medic or an external doctor (PN 2, 3, 8, 9, 17).

Those involved in Gaelic football, camogie, hurling, soccer, and other sports lacked access to such resources. However, they still encouraged injured athletes to request relevant tests through their general practitioner or private physician. As PN 13 explained, these tests offer a “*true picture of what was actually going on physiologically*” compared with relying solely on reflective screening, asking questions like “*What are you eating? What’s your training load?*” in isolation. PN 5 added that understanding the athlete’s internal physiology provides PDs/PNs with tangible “*information”* they can *“work from*” design tailored nutrition strategies.

Blood assessments were frequently cited as instruments in identifying deficiencies that could negatively impact athletes’ performance, recovery, and injury outcomes. For example, simple blood tests among female rugby athletes flagged that their “*ferritin levels*” were “*in their boots*” (PN 3), suggesting poor adherence to dietary advice. PN 9 noted, despite athletes claiming to eat “*two or three steaks a week,*” test results suggested otherwise. PN 3 shared that they routinely screened their female rugby players at start of the season, corrected any deficiencies, and “*move on*”. Other bloodwork helped identify masked affecting estrogen levels, bone health, and feeding, leading to osteoporosis and severe fractures (cited below) (PN 14).

“*Her oestrogen was the same as postmenopausal women… she needed to understand the link between oestrogen and bone protection. She needed to understand the link between under fuelling and potentially cortisol production, and its secondary link to bone health... to understand how her bloods were… how her previous injury profile had been leading to this massive fracture*.” (PN 14)

Participants (PN 13, 14) used dietary tools to assess eating behaviors and identify athletes at risk of injury or suboptimal nutrition. The LEA questionnaire revealed that some male cyclists were unintentionally “*undereating by the guts of 1000, 1500… up to 3000*” calories per day (PN 13), highlighting the need for fueling education. All PDs/PNs reported using ≥1 method of dietary assessment, such as food frequency questionnaires, 3-d diet diaries, or 24-h recalls, at various points in the season and post injury. Some participants used tracking app like MyFitnessPal (Under Armour, Inc.) and, or Nutritics (Nutritics Dietary Analysis Software) (PN 4, 10, 13) to both educate athletes’ and monitor their eating behaviors. PN 13 emphasized the role of energy availability in maintaining physiological health and recovery, noting that Nutritics helped identify nutrient “*markers going up and up to reach their levels…”* post injury (PN 13). PN 5 described an “*awakening*” when reviewing food diaries, identifying that group messaging was not effective with Gaelic athletes and targeted education was needed. For them, “*energy availability and carbohydrate availability”* emerged as “*more important to focus on”* than initially perceived (PN 5). Suboptimal “*calcium and iron*” intakes also presented as some athletes, influenced by social media and “*certain documentaries*” began reducing their consumption of “*animal-based products*” (PN 5).

These screening assessments provide a clearer picture of athlete’s current health and nutritional status, highlighting areas that need focus to facilitate healing and recovery. However, consistent use is essential to build meaningful longitudinal data on athletes’ health and injury history. Participants who used these assessments cited that athletes would frequently present with deficiencies in calories, macronutrients (CHOs, fats, and proteins) and key micronutrients such as calcium, iron, both before and after injury events.

PDs/PNs cited that undereating CHO and “carb phobia” as common issues across the board in athletes involved in rugby, field-based, and endurance sports [64.7%, *n* = 11/17; (PN 2, 3, 5, 6, 7, 9, 10, 11, 13, 14, 15, and 16)]. PN 7 observed that young and older male Gaelic footballers were “*carb-phobic*,” and this became more pronounced for them post injury, with athletes “*cutting calories right across the board, not realising that being in a large energy deficit is going to delay their recovery.*” PN 14 cited mixed messaging as the issue, demonising CHO, and stating,

“…*it is actually worse than it was because the focus is very much on protein and not carbohydrates*… *There is… a tendency for a leak of the advice for rugby into GAA, and they are not the same game at all*” (PN 14).

In comparison, rugby athletes, “*based upon of any analysis of any diet diaries”* under-fueling was less of an issue (PN 2). Endurance athletes, however, tended to avoid fats and over-consume simple CHO habitually.

“*a lot of cyclists are middle-aged tend to maybe overdo the carbohydrates, and underdo the fat, and trying to have the conversation around fats, that they don’t make you fat, do you know this? That has been a struggle to kind of get their heads around… those are the ones who tend to be incurring more hip-based injuries; I’ve been finding, hip and knee…”* (PN 6)

### Nutrition management

#### Energy availability and macronutrient management

First and foremost, PDs/PNs prioritize managing athletes’ energy availability to prevent a negative balance occurring acutely post injury. As PN 16 mentioned, inadequate energy intake from “*not eating enough*” can increase muscle protein breakdown and accelerate tissue loss.

“*But, obviously*… *if they don’t have their energy right, then that’s going to take up a large part of that energy balance... So, you can see how trying to balance the overall total energy intake with the actual protein macronutrient intake…*” (PN 16)

Some PDs/PNs described setting appropriate calorie targets to meet the increased demands of the postinjury period, address suboptimal macronutrient intake, and support elevated energy expenditure resulting from allostatic stress and tissue healing.

“*Especially in that initial phase of injury, we really tried to kind of get in there to, to remind them, well actually this is a really energetically demanding. So, trying to keep calories up and some might struggle with appetite. So, we really try just focus on like liquid calories or you know, smoothies and milks… try keep those calories up, and support kind of the kind of healing stage.*” (PN 8)

To assess overall energy intake, PDs/PNs reported using food diaries, 24-h recalls, and 3-d diet records, often comparing pre- and postinjury data. As PN 5 explained, they **“***look at the quality of the food”* and may use “*a three-day diet diary or 24-hour recall or maybe both”* (PN 5). On the basis of these assessments, practitioners adjusted caloric intake to support maintenance or slightly elevated targets to promote tissue healing. However, participants did not explicitly report the use of formal energy availability equations or validated estimation tools [e.g., kcal/kg fat free mass (FFM)/d] to guide nutritional interventions during injury recovery [1,27].

A majority of PDs/PNs (70.6 %, *n* = 12/17) advocated a “*food-first*” approach, emphasizing the inclusion of nutrient-dense, whole, minimally processed foods that injured athletes “*actually enjoy*” (PN 15). Figure 4 illustrates nutrition management strategies shared by individual PDs/PNs, highlighting consistent postinjury energy and protein targets, along with the promotion of micronutrient intake. However, nuanced challenges also emerged, as noted by PN 11:

“*The first girl that I worked with [1-1]… I think maybe four of those monsters a day was kind of what was keeping her going… She didn’t really eat much other than that. It was actually amazing to see within a short… couple of months the difference. I suppose eating food is always helpful anyway, isn’t it? As opposed to drinking caffeine and sugar?”* (PN 11)

Additionally, participants highlighted considerable variability in their athletes’ food behaviors and mindsets post injury. For example, some athletes “*cut down the amount they are eating because they think, ‘I’m going to put on a lot of body fat if I am not doing anything’, so, ‘I don’t want to be eating a lot of food’”* (PN 16).

Furthermore, poor energy availability impacted athletes’ macronutrient intake and subsequent utilization. CHO management was cited by 58.8% of PDs/PNs [*n* = 10 (PNs 2, 4, 6, 7, 9, 11, 13, 14, 15, 16)], involving minor reductions based on athletes’ postinjury expenditure. The remaining 41.2% [*n* = 7 (PNs 1, 3, 5, 8, 10, 12, 17)] did not cite specific adjustments. Strategies prioritized CHO quality over quantity, with PDs/PNs advising against drastic reductions. Instead, they recommended a shift toward fiber-rich, complex CHO sources (e.g., whole grains, fruits, and vegetables) to align with reduced activity levels, and support recovery. The aim was for a “*more careful*” CHO selection in the early stages (PN 4).

“*…not reducing carbs too much because you need them to live… maybe the type of carbohydrates, high, high fibre carbs so that they are not reducing carbohydrate completely…*” (PN 4).

Additionally, postinjury fat intake was advised to include ω-3 fatty acids and foods rich in unsaturated, while being mindful of saturated fat, and ω-6 intakes.

Early nutrition support and communication within 2–3 d post injury was deemed vital, along with being attuned to an athlete’s mental state during this time, to help refocus food behaviors. For example, PN 9 encouraged their injured athlete to shift priorities saying, “*I don't want you to get lean during this stage; it’s not about your body composition; it’s about your healing*.” Giving their athlete permission to relax dietary rigidity, allowing “*more error, room for error*” as a slight increase in body fat (e.g., 5 or 10 mm) is more beneficial than harmful in the early stages of recovery (PN 9). However, PDs/PNs also stressed the need to maintain close, open communication to ensure athletes feel supported and avoid overindulging down the “*pizza and beer route*” and neglecting nutrition and negatively impacting their recovery (PN 9).

Conversely, some athletes were cited as having resisted support, going “*off the radar*,” or lost motivation, especially if their season had ended, leading to less focus on their recovery. PN 9 shared an example of an athlete who sustained a lower limb fracture during the final tournament of the World Cup, went “*off the radar*,” went on holidays, and returned *“5 kilos”* lighter, yielding *“18 months”* recovery to regain full function (PN 9).

#### Protein intake

The next focus mentioned by all PDs/PNs was ensuring adequate protein intake to support recovery, as it helps “*preserve lean body mass*” (PN 3) and maintaining “*muscle mass as much as possible*” (PN 13) in injured athletes. All practitioners increase athletes’ protein intake post injury. Five participants [29.4%, *n* = 5/17 (PN 2, 6, 10, 16, 17)] recommended minimum intakes of 1.2–1.6 g/kg/d, with upper targets ranging from 2.0 to 3.0 g/kg/d [1,3,44,45]. For example, PN 16 advised 1.2–2.0 g/kg/d, but acknowledged that “*research would suggest… anywhere from 2.0 to 2.5 as a good range for someone who’s injured”* (PN16) [1,3,27,30,45]. PN 2, 10, and 17 similarly recommended intakes of 2.0 g/kg/d or greater. PN 6 advised 1.6–1.8 g/kg/d in healthy states, increasing post injury to “*between 2.0 and 3.0 grams per kilogram,”* depending on the athlete’s body mass (PN 6) [1,3,30,45].

Furthermore, 29.4% of participants [*n* = 5/17 (PN 4, 6, 7, 13, 17)] advised consuming 20–40 g per protein servings spread evenly across 4–6 meals or snacks throughout the day to meet these targets (Figure 4). Although PN 3 mentioned “*protein pulsing*” of 10–15 g every 2 h, for acute bone healing. Consistent protein intake was emphasized to ensure athletes are “*getting their protein in the morning, afternoon and evening, probably four to five servings per day”* (PN 17). More specifically, 3 PDs/PNs mentioned the importance of “*the amino acid profile*” of food, especially the essential amino acid leucine (PN 6, 16, 17), for its role in tissue repair and recovery [1,3,27,[30], [44]]. For example, adding “*yogurt*” to smoothies and casseroles as a convenient source increases the leucine content of meals (PN 6). Although protein “*buy-in*” from athletes is generally high, PDs/PNs focus on supporting inclusion of a variety of high-quality animal and plant-based protein sources, including protein powder. PN 6, 7, 9, and 16 state that this variety helps “*flicker*” protein synthesis rates and supports tissue repair.

#### Nutrient density and micronutrient intake

Next, PDs/PNs (70.6%, *n* = 12/17) remind athletes to boost their intake of micronutrient-rich, antioxidant, and anti-inflammatory foods postinjury. They encourage athletes to add 2 different colored fruits and/or vegetables to ensure “*plenty of colour on their plates*” (PN 17), promoting nutrient variety and helping athletes achieve “*seven plus daily*” servings (PN 8). PDs/PNs also support adding ω-3-rich foods to enjoyable, easy-to-prepare meals such as smoothies and burrito bowls. Additionally, PDs/PNs (PN 6, 10, 11) help athletes reduce their intakes of ω-6 fatty acids, highly processed foods, and takeaway meals to further aid healing.

“*…are we consuming takeaways? It*’*s probably like the first thing we want to try to eliminate. We want to try and get as much kind of leafy green veg and good quality vegetables and good quality just balanced nutrition into their diet.”* (PN 10)

Supplements are typically recommended when a deficiency or nutrient gap is identified, most commonly vitamin D_3_ during the winter months in Ireland (70.6%, *n* = 12/17). However, specific clinical deficiency thresholds were not reported [48]. Dosages cited by PN 6 and PN 10 dosages ranging from 2500 to 3000 IU/d, aligning with research suggesting intakes between 2000 and 4000 IU/d [45,48]. Multivitamins (29.4%, *n* = 5/17) and ω-3 fish oil supplements (58.8%, *n* = 10/17) were used if athletes did not eat oily fish, were “*pickier eaters,*” or unable to meet micronutrient requirements through food alone. For injury, PN 6 and 12 recommended from 2 to 3 g daily [[46], [48]]. Beyond addressing injury-specific needs, these supplements were also used by PDs/PNs as a standard to optimize athletes’ recovery and overall health.

“*So maybe they are somebody that doesn’t consume a lot of fruit, veg. Can we look at that and increase that to manage the inflammation? I suppose again, depending on the injury, we would look at, say, certain supplements…*” (PN 17)

However, 82.4% (*n* = 14/17) cited that a well-balanced diet using a “*food first*” approach was “*a good rule of thumb,*” especially in the acute phase post injury, to prevent deficiencies in fundamental nutrients (PN 16). Even though,

“…*there is mixed research behind various different, let’s say, vegetables or even fruit with regards to inflammation and antioxidants. We're kind of like it's not going to do any harm! We try to put these messages forward causse it’s going to benefit their immune system* anyways…” (PN 8).

#### Nutritional strategies to support stages of tissue healing

**FIGURE 5:**
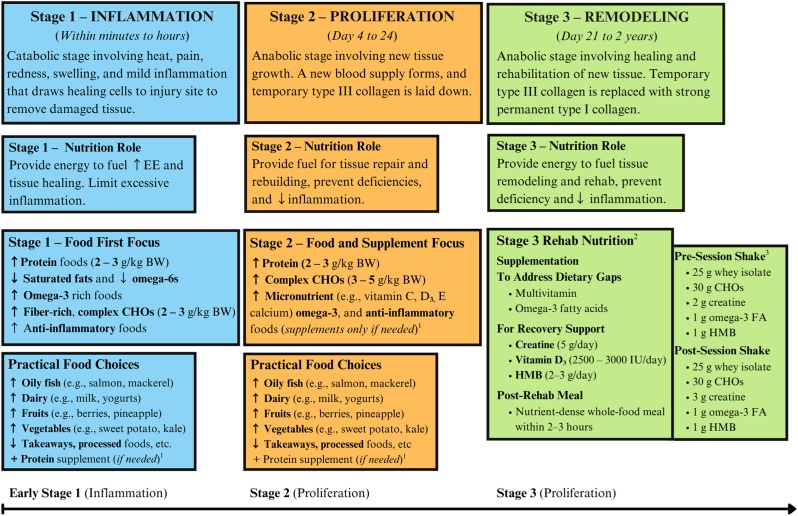
Postinjury nutrition strategies mentioned by performance dietitians and nutritionists, aligned with the physiological healing stages of injured tissues. Nutrition needs were stage specific. The figure outlines stage, timeframe, nutritional role, key focuses, and practical applications. Priorities include energy, protein, nutrient-dense foods, and targeted supplements. All cited ranges are examples from PDs/PNs practice and should be considered on a case-by-case basis. +, add; BW, body weight; CHO, carbohydrates; EE, energy expenditure; FA, fatty acids; HMB, β-hydroxy β-methylbutyrate; IU, international units; MPS, muscle protein synthesis; N, nutrition; PD/PN, performance dietitian/nutritionist; RED-S, relative energy deficiency in sport; S, stages of tissue healing (S1, S2, S3); ↑, increase or surplus; ↓, decrease or reduce. ^1^Protein supplementation may be used during all stages to support meeting elevated protein needs. However, no additional supplements are recommended in stage 1. In stage 2, supplementation may be considered on a case-by-case basis to address dietary gaps. Protein should be ≥1.2–1.6 g/kg/d, with an upper target of 2.0–3.0 g/kg/d [1,3,27,30,44,45]. ^2^Stage 3 includes a structured rehab nutrition protocol that combines food-based and supplement strategies. ^3^Stage 3 training nutritional protocol cited by 1 participant (PN 6).

This category was not strongly discussed among PDs/PNs. However, 3 participants [17.6%, *n* = 3/17 (PN 6, 9, 16)] provided detailed insights, delving into the “*nitty gritty*” postinjury nutrition strategies aligned with physiological healing stages of injured tissues (Figure 5) [1,3,27,30,44,45].

Stage 1 involves managing inflammation but cautions against “*jumping in and trying to do every single thing to reduce that inflammation*” acutely, as it may not be *“the best thing to do”* (PN 16). The consensus among PDs/PNs was to monitor and manage athletes’ inflammation while using a “*food first,*” as previously mentioned, to increase their intake of antioxidant-rich foods and prevent inflammation from worsening. Figure 5 presents strategies cited for stage 1, which include increasing protein targets of 2–3 g/kg of body weight (g/kg BW), reducing intake of proinflammatory foods such as saturated fats and ω-6 fatty acids, and increasing foods rich in ω-3-rich (e.g., flax seeds and oily fish) [3,30,45,48]. CHO intake is adjusted according to training stimulus and energy expenditure for injured athletes. PDs/PNs encourage more complex, and fiber-rich types of CHO sources, such as “*fruits and their veggies and their whole grains*” (PN 6). Although supplements are not typically mentioned during this stage (PN 6), whey protein was cited as a helpful food-based option to meet elevated protein targets and to “*get good amino acids into the diet*” (PN 6) particularly when achieving these targets though food sources alone may be challenging.

Stages 2 (proliferation) and 3 (rehabilitation and remodeling) require adequate energy availability to support elevated metabolic demands and to fuel the rebuilding and strengthening of newly formed tissue. PDs/PNs cited that athletes often attempt to cut calories or experience reduced appetites due to their injured state, immobility, and decreased training levels. This behavior forces the body to draw on lean tissue reserves for energy, impair metabolic function, and ultimately delay recovery.

“*Depending on the type of injury, dependent severity, injury, a lot of injuries*… *increase energy expenditure at the local level so they may not have to drop calories as much as they think that they do… So, if they’re already in a deficient state, and then they drop them(calories) even further… they could possibly be delaying the recovery process or at least being suboptimal*” (PN 16)

Stage 2 (days 4–24) involves more targeted nutritional strategies. Athletes are encouraged to maintain elevated dietary protein intake and consume 3–5 g/kg of complex CHOs (PN 6), consistent with current rehab and recovery recommendations [3,48]. They are also advised to increase their intake of micronutrient, antioxidant, and anti-inflammatory-rich foods (e.g., fruit, vegetables, and oily fish) to support adequate levels of key nutrients, including vitamins C, A, E, and D_3._, and minerals magnesium, and calcium. At this stage, PDs/PNs cite that supplements may be considered to prevent deficiencies, with multivitamins, creatine, and collagen cited (PN 6, 9) [3,27,45].

“*I won*’*t supplement until stage two, which could be after four days I’d start to bring supplements in… I encourage them to get as much of their foods or their nutrients coming in from whole food sources ehmm, and then with extra super dosing of the supplements from there”* (PN 6)

Stage 3 (rehabilitation and remodeling) spans from day 21 up to 2 y post injury [[4], [5],^27^]. PDs/PNs shift to focus on helping tissue regain capacity, strength, and function to minimize reinjury risk (PN 6, 9). As athletes progress through rehabilitation and new tissues form, their metabolic rate increases, requiring higher calorie intakes than in stage 1 or during sedentary periods. PDs/PNs cite adjusting calorie and CHO intake to match the demands of their athlete’s rehabilitation plans [35.3%, *n* = 6 (PN 2, 6, 8, 9, 12, 17)]. The “…*body needs a huge amount of energy for injury rehab”* to support effective healing within a limited timeframe (PN 15). Caloric needs are very individual “…*if they are healing quite fast, and they*’*re starting to get more activity in, and their requirements are going up as a result”* (PN 6). Likewise, further adjustments in CHO intake will be made if healing accelerates or more activity is introduced.

For stages 2 and 3 of injury recovery, PN 6 highlighted the use of supplements such as creatine, β-hydroxy-β-methylbutyrate (HMB), whey protein, and CHO as essential components. This participant described a supplementation protocol based on [30,45], with specified doses and timing intended to target optimal “*windows*” of increased blood flow to injured tissue during or after sessions. The protocol includes both pre- and posttraining or rehabilitation shakes: the preshake contains 25 g whey isolate, 30 g CHO, 2 g creatine, 1 capsule of ω-3 fatty acids (1 g), and 1 g HMB; the postshake includes 25 g whey isolate, 3 g creatine, 1 capsule of ω-3 fatty acids (1 g), and 1 g HMB, followed by a nutrient-dense meal ∼2 h later (Figure 5 and Supplemental Figure 1).

It is important to note that this supplementation strategy was reported by only one of the 17 performance nutritionists interviewed; therefore, it represents a limited snapshot rather than a common practice among participants. However, detailed protocols may be helpful, as the postinjury period is often psychologically challenging. Athletes’ motivation and “*buy-in*” strongly dictate recovery and tailored strategies may support engagement by providing reassurance and structure, ticking,

“*…the positive box; the person might be more or less interested; they might have more or less money… you just throw out all the options for them.*” (PN 5)

Or unknowingly supporting recovery *“…eating two kiwis before bed, that’ll really improve your sleep!*” (PN 13). Some PDs/PNs advised a range of options once the “*basics*” were met *“…because you're trying to get any of those little percents*” (PN 12). Though, at times, it was clear they did not wholeheartedly believe in them, *“I’m not saying it will work, but… like, you might as well just take it*” (PN 12).

However, behind all PDs/PNs strategies, having an understanding of their audience, that is, the injured and ensure their support “*needs to be more practical*” than overly technical. Instead of *“talking about polyunsaturated and monounsaturated fats and… trying to get”* athletes to understand these details, simplicity is key, as athletes “*lose it… even the best of them*.” Focusing on diet quality with “*more colours, more fruit and veg; 5 to 7 portions a day*” will be more worthwhile (PN 4).

## Discussion

This study explored how Irish PDs and PNs assess and manage athletes’ nutrition immediately after injury, adapting their strategies based on contextual factors such as injury type, sport resourcing, and multidisciplinary collaboration. Findings indicate that nutrition support varies significantly across settings, primarily influenced by PDs and PNs’ timely awareness of injury onset. This awareness depends on effective team communication, organizational structures, practitioner autonomy, and the value placed on nutrition within the sport environment.

During the acute stage (injury onset to 7 d), early identification enables PDs and PNs to implement targeted nutrition strategies to support tissue healing. Initial efforts focus on comprehensive athlete profiling through health screenings and nutrition assessments, which subsequently guide ongoing practices throughout tissue healing, injury recovery, and RTP.

Noncontact-related injuries were commonly associated with LEA and deficiencies in key nutrients such as CHO, iron, and vitamin D. These insufficiencies increased physiological stress, elevated the risk of RED-S, reduced immunity, and hindered tissue repair.

Interviews revealed the need for practical, easy-to-implement nutritional guidelines for PDs’ and PNs working in resource-limited setting (e.g., limited staff, tools, funding). Discussions highlighted a desire for adaptable early intervention frameworks that could accommodate diverse sports, injury types and tissue healing demands, while remaining flexible enough to for nuanced sporting scenarios.

Although many practitioners used a general framework to ensure energy availability and adequate protein intake from nutrient-dense foods, approaches to early healing and recovery varied. Some practitioners tailored their strategies to the athlete’s specific tissue healing stage, injury severity and rehabilitation plan.

Early-stage nutrition management was often influenced by communication, collaboration, and resource constraints. When dietary intake was insufficient, multivitamin supplements were introduced to address gaps (e.g., low fruit and vegetable intake), prevent deficiencies (e.g., vitamin D_3_), and support immunity during periods high allostatic stress and metabolic demand [3,27,47,48]. Supplementation was considered when dietary solutions were not enough. However, participants did not reference clinical thresholds (e.g., vitamin D_3_, serum 25(OH)D < 30 nmol/L for vitamin D deficiency [27], an ω-3 index > 8% [46], and ferritin < 30 μg/L) [49] to guide their decisions. Regular monitoring of micronutrient status is advised to ensure safe targeted use [3,27].

Some, PDs/PNs incorporate additional supplements, such as creatine and collagen, to reduce atrophy and support tissue repair and rebuilding [1,3,27]. These were introduced based on factors including tissues healing stage, tissue type, injury site, severity and athlete mobility or training capacity [[3], [4]]. Specifically, stage 2 and 3 involve replacing the newly formed temporary type III collagen tissue (which resembles cooked spaghetti) with stronger, more durable type I collagen tissue that mimics and restores the original tissue’s function [50].

Stage 3, the most prolonged recovery phase, spans from day 21 up to 2 y post injury, focusing on rehabilitation and tissue remodeling [[3], [4],^27^]. During this time, PDs/PNs shift emphasis to restoring strength and functionality to ensure athletes are supported through their gradual RTP. Ongoing monitoring and assessment are essential for adjusting nutritional strategies to support recovery and prevent reinjury. Furthermore, support should not be siloed; collaboration among the broader backroom team, including PDs/PNs, strength and conditioning coaches (S&C), and physiotherapists, is crucial for tailoring rehabilitation plans and addressing athletes’ evolving needs throughout their recovery.

The effectiveness of nutritional support was heavily influenced by practitioners’ awareness of injuries and quality of communication within the support team. Communication quality shaped subsequent planning and management strategies for tissue healing.

Gaps in injury management emerged, driven by team environment, limited understanding of PDs’/PNs’ roles, educational needs, funding constraints, athlete burnout, under-fueling, and variable attitudes among athletes and coaches. These issues are linked to poor communication and breakdowns in acute injury care. Acute injuries generally have a sudden onset from a specific event [4,5].

Practitioners’ prior exposure to sports injuries within their setups greatly influenced their approaches to nutrition management. Differences emerged based on communication quality, sport type, gender dynamics (e.g., women’s rugby, ladies Gaelic football, and soccer), individual compared with team athlete contexts, and competition level. Rapport between PDs/PNs and athletes was also crucial. Full-time practitioners or those with consistent one-on-one engagement often delivered more timely and specific nutritional interventions than part-time practitioners, particularly in nonelite environments, where communication was less direct.

External factors, including athletes’ and support staff’s attitudes and behaviors, influenced practitioners’ injury awareness. Effective communication enabled PDs/PNs to support tissue healing and recovery by extending nutrition strategies beyond the traditional focus on performance and body composition. Despite employing a range of nutrition strategies, significant differences were evident in how injuries were initially communicated to PDs/PNs and in the subsequent nutrition support provided.

### Communication and its impact on injury awareness and nutrition support

Effective communication and collaboration among athletes, PDs/PNs and the broader backroom team are essential for timely injury recognition, coordinated care and delivery of evidence-informed recovery and nutrition strategies [18,28,[51], [52], [53]]. This study reinforces communication as a cornerstone of injury management, enabling PDs and PNs timely, targeted interventions.

Prompt, transparent dialog between athletes and practitioners facilitates early nutrition strategy implementation aligned with injury onset, healing, and recovery stages [3,27,28]. Conversely, communication breakdowns, particularly within team sport contexts; delayed PDs’ and PNs’ awareness of injuries, limiting them in providing timely nutritional support. Delays, often due to internal processes or hierarchies, were a source of professional frustration and, in some cases (e.g., PN 4), strained backroom relationships.

Communication access varied by PDs’ and PNs’ roles. Full-time embedded PDs/PNs (e.g., PN 2, 5, 8), particularly in elite team sports such as rugby, and Gaelic football, benefited from direct communication and structured injury notification systems. However, even in these more superior settings, integration was inconsistent, with some PDs/PNs (e.g., PN 9, PN 14) reporting delays of ≤2–10 d due to communication hierarchies.

Part-time practitioners (e.g., PN 4, 7, 15) had less formal access, relying on informal check-ins, working outside contracted hours, or positioning themselves near physiotherapists. These workarounds reflected proactive intent but ultimately delayed their athlete engagement and reduced their ability to optimally influence acute recovery strategies. In contrast, PDs/PNS working directly with individual athletes (e.g., PN 6, 16, 17), whether private practice or full-time, typically had earlier injury awareness due to their stronger athlete rapport, enabling timely, tailored nutrition support and better adherence. However, limited integrated in amateur or self-managed athlete contexts, restricted PDs/PNs capacity to coordinate broader recovery plans and multidisciplinary care.

Across contexts, injury management offered PDs/PNs opportunities to build trust, educate athletes, and adapt nutrition to evolving needs. However, practitioner accessibility was crucial, enhancing relationships, athlete empowerment, and psychological safety during recoveries [26]. Communication and collaboration were stronger in team setting. Structural integration, accessibility, and quality of athlete–practitioner relationships influenced feasibility, timing, and individualization of injury nutrition support. Although individual athlete PDs/PNs delivered more tailored interventions, limited support systems caused communication delays, impacting timely and practical stage-specific nutrition implementation.

Poor collaboration within the backroom teams has been linked to increased injury burden and reduced player availability in elite football [52,54]. In the Irish context, weak communication and limited access to interdisciplinary support reduce athlete trust, delay injury disclosure, and hinder RTP decisions [18]. Without timely updates, PDs/PNs are unable to implement appropriate nutrition interventions, particularly given the frequent exclusion of nutrition from RTP models [51,52,55].

These communication factors shaped not only the timing of nutritional interventions but also the feasibility of implementing stage-specific strategies throughout the recovery process.

Teamwork off the pitch is as important as on it [28,[55], [56]]. Enhancing communication channels and fostering openness among PDs/PNs, coaches, medical staff, and other support staff can significantly improve recovery outcomes for injured athletes.

Our findings align with research emphasizing the value of interdisciplinary collaboration in injury recovery [55]. However, inconsistencies in nutritional strategies point to a disconnect between scientific knowledge and its application in practice [28,32,56,57]. All participants held postgraduate qualifications in nutrition and exercise science, but practice was shaped more by their sporting context and experience than formal education. This highlights the need for a practice-informed injury management framework in sports nutrition [53].

The absence of nutrition-specific injury management protocols was particularly evident when examining recovery timelines. Although PDs/PNs recognized distinct injury phases (e.g., acute, e.g., PN 6, 16), their ability to implement precise, stage-specific nutritional interventions varied due to communication challenges [4]. This variability contrasts with evidence-based sports nutrition recommendations, which emphasize targeted strategies such as increased protein intake for muscle repair and specific micronutrients support for collagen synthesis [4,27,30,45].

### Impact of back-team collaboration

Effective communication and collaboration among backroom practitioners and athletes are critical for delivering timely, comprehensive recovery strategies [28,52]. Athlete-centered injury recovery depends on open communication and clear role recognition within the interdisciplinary teams. Optimal injury management relies more on cohesion, role clarity, and collaborative care within the back-team structure than by its size [54,58].

The back team typically includes PDs, PNs, psychologists, medical doctors, physiotherapists, athletic therapists, S&C, and sports scientists working cohesively, with player health as the central focus [18,28,55]. In Irish sport, communication gaps and resource variability (e.g., by gender, level, and sport type) can limit the effectiveness of nutrition support. To address these barriers, standardized education and workflows for injury-related nutrition care are essential to ensure consistent, athlete-centered support.

In environments with strong communication channels, full-time PDs and PNs working with individual athletes or within semiprofessional and elite-level teams were well informed about injury events, severity, and recovery timelines, enabling timely and appropriate nutrition support. However, in team-based or amateur sports where nutrition support was a lower priority, PDs/PNs reported limited communication and minimal, if any, opportunities for athlete profiling [28,59]. When included in the decision-making process, PDs/PNs were better position to deliver effective nutrition care. In settings with suboptimal communication, PDs/PNs must carefully balance collaboration with the need to assert their professional role. Strengthening their autonomy through evidence-based practice, staff education, and relationship-building will help embed nutrition more firmly in injury care pathways [53,55].

All members of the support team share a collective duty of care and must collaborate to prevent breakdowns across key health pillars during injury recovery, RTP timelines, and the broader performance journey [[28], [66]].

Despite increased availability of practitioners through in-person contact and online platforms (e.g., WhatsApp), some athletes underreported injuries due to a perception that their PD/PN may be overstretched [60]. This concern may be valid, as 47.1% of participants in this study held multiple part-time roles and reported feeling undervalued or misunderstood within their settings. Although access to PDs/PNs remains a key limitation within Irish sport, placing athletes at increased risk of inadequate nutrition knowledge and suboptimal dietary intakes, thereby compromising injury management support [32]. Female athletes in amateur contexts reported receiving less comprehensive nutrition support compared with their male counterparts [[13], [28],^66^]. In these settings, PDs/PNs reported added challenges in ensuring adherence to nutritional plans, often exacerbated by funding limitations, and less structured team environments, and in-person contact time.

### Barriers to nutrition management

Several barriers to optimal nutrition management emerged from participant interviews, primarily due to communication breakdowns between PDs/PNs and other members of the support team, including coaches and medical personnel. Additional challenges included athlete burnout and under-fueling, particularly among part-time practitioners working with young athletes (PN 7, 17) and those supporting female soccer players (PN 11) where meeting athletes’ nutritional needs during recovery proved difficult. Sport-specific factors, such as attitudes toward nutrition (e.g., cutting calories, carb phobia) and hyperfocus on body composition when the body requires nutrients for healing further hindered adherence to recovery protocols and delayed tissue healing.

Sports nutrition research supports stage-specific strategies to enhance tissue repair, reduce muscle atrophy, and improve RTP outcomes [3,13,27,30,61]. However, limited knowledge and resource constraints hinder implementation in Irish sporting contexts [28,[59], [66]]. These barriers affect both athletes and PDs/PNs, compromising injury management and recovery outcomes [32].

### Recommendations and practical implications

This study reveals an urgent need for practical, evidence-based nutritional guidelines to support injury management in Irish sport. Drawing on the experiences of Irish PDs and PNs, the findings offer valuable insights into enhancing early communication, interdisciplinary collaboration within backroom teams, and systematic athlete profiling and screening. These elements are foundational to effective injury management within the unique context of Irish sport.

Although evidence-based strategies for initial screening and injury nutrition do exist [3,27], critical gaps remain in their practical application. These gaps are largely due to limited access to PDs and PNs across Irish sports settings [13,32]. At both organizational and sport-specific levels, this lack of access contributes to inconsistent and often unreliable nutritional support from a range of practitioners who influence athletes [32,57].

Addressing these gaps is essential to safeguard athletes’ health and support sustainable performance throughout all phases of recovery. Access to expert guidance from qualified PDs and PNs is crucial to raising awareness of nutrition for injury management and delivering context-specific, evidence-informed care [[13], [28],^57^].

To address systemic barriers, policy-level action is required. National governing bodies (e.g., Sport Ireland, the IRFU, the GAA) must collaborate to mandate access to qualified PDs/PNs across all levels of Irish sport. Nutrition services should be embedded within athlete support programs, with dedicated funding for nutrition roles and the integration of injury management nutrition education across all disciplines. Standardizing access in less resourced or amateur settings is essential to ensure unbiased injury support and recovery outcomes. There is also a fundamental need to develop and implement evidence-based nutrition guidelines that are adaptable to the dynamic, and unpredictable settings in which Irish PDs and PNs operate. Structured communication protocols within backroom team could improve injury awareness, facilitate earlier nutritional interventions, and ultimately improve recovery outcomes and while reducing injury burden.

Nutritional strategies should be aligned with the stages of tissue healing [27]; however, few practitioners are currently equipped to apply stage-specific strategies effectively. Multidisciplinary collaboration is essential to support athletes’ recovery and should involve all relevant sports practitioners in designing plans that address the energetic, nutritional, and psychological demands of injured athletes [55]. Nutrition plays a crucial role modulating allostatic load, which influences tissue overload and repair [1]. Consequently, targeted nutritional interventions are essential throughout rehabilitation to support energy-intensive processes involved in tissue regeneration and repair, such as protein synthesis, and cytoskeleton remodeling [27].

Incorporating an adaptable, evidence-based framework like the “Ask, Acquire, Appraise, Apply, Audit” model [53], may improve consistency in practitioners nutritional care while accommodating the often-chaotic nature realities of sporting environments. Athlete profiling and injury prevention protocols, including nutritional screening, should be integrated into routine seasonal assessments to identify risks before injury occurs. In lower resource settings, simpler alternatives (e.g., ISAK measurements instead of DXA scans) may be a more feasible lower cost best-practice approach [63].

Effective communication and timely injury awareness are critical for PDs and PNs to support athletes’ recovery and RTP but depend on the attitudes and behaviors of athletes and support staff toward nutrition, especially during injury. Beyond attitudes and perceptions, all participants are performance nutrition professionals with postgraduate degrees (MSc/PhD) in nutrition, sport, and exercise, providing strong theoretical knowledge to manage injuries based on scientific research. However, their practical expertise is acquired from hands-on experience, adapting and applying nutritional strategies within real-life, high-pressurized sports settings.

PDs and PNs must prioritize injury nutrition strategies that are timely, practical, and easy to implement. Despite external pressures to expedite RTP decisions, athletes’ health should take precedence over performance metrics. Participants shared various initial injury nutritional strategies tailored to individual needs, highlighting the need for both specificity and flexibility in nutritional planning. These practice-informed strategies go beyond epidemiological or biological understandings of injuries [[4], [5]], offering real-world nutritional strategies that inform both practitioners and researchers. Practice-informed nutritional strategies enhance athletes’ performance, prevent deficiencies, and support effective tissue repair and healing [1,28]. Despite growing demand, there remains a clear lack of injury-specific, evidence-based nutritional guidelines. Developing the best-practice protocols is essential. An adaptable evidence-based framework, such as *Ask, Acquire, Appraise, Apply, Audit* can provide PDs and PNs with accessible, needs-based tools suitable for the unpredictable and dynamic nature of Irish sporting environments [9,18,32,53,[62], [64], [66]]. In addition, future research should explore emerging biomarkers to measure athletes’ workload, injury risk, acute tissue damage, and inflammation [[61], [65]], as well as the implementation of nutritional interventions such as collagen, creatine, and ω-3s [1,28,55] and assess their application across applied athlete populations and Irish sporting contexts.

### Limitations

The findings of this research reflect a specific point in time and should be interpreted within the context of an evolving field. As performance and injury nutrition practices advance with emerging scientific evidence, some identified factors in this study may become less applicable in the future. Although the sample size was relatively small (*n* = 17), it is representative of the Irish cohort of PDs/PNs and offers valuable insights into current, real-world practices within Irish sport. These findings are relevant for sport PDs, nutritionists, coaches, and other practitioners involved in managing sports injuries. The sample included practitioners from a wide range of sporting bodies (Table 1), with strong representation from national governing bodies such as the GAA and IRFU. This reflects the organizational structure of professional and Gaelic sports in Ireland; however, it may limit the applicability of findings to PD/PN practices within less formalized or resource-constrained sporting environments. This research highlights current best-practice nutritional strategies and proposes protocols for PDs and PNs to streamline athletes’ initial injury management. It also identifies research gaps that affect PDs’ and PNs’ decision-making, which in turn influence the implementation of management protocols and athlete adherence.

A key limitation is the need for more education or formal training specific to the role of PDs/PNs in sport during acute injury management, compared with other professionals such as physiotherapists. Participants demonstrated varying levels of understanding regarding the potential role of nutrition in injury recovery strategies. The sporting environment, particularly within teams and professional setups, significantly influenced participants’ awareness, diagnosis, and management of athletes during the immediate, acute injury phase. Additionally, attitudes and cultural norms differed across sports, competition levels, and team settings. Resource availability for athletes was another critical factor, with clear differences observed between elite and amateur sports as well as male and female teams. These variations affected PDs’ and PNs’ awareness of injury events, and the level of nutritional support provided during the initial stages of recovery. Additionally, limited access to assessment tools constrained PDs and PNs nutritional management strategies.

All interviews were conducted and analyzed by the lead author, with bias mitigated through reflexive journaling and peer review. Although manual coding in Word and Excel offers less streamlined traceability than qualitative software, thorough documentation, enabled deep, iterative engagement with the data.

In conclusion, this study provides critical insights into how Irish PDs and PNs manage nutrition during the early stages of injury. The quality and timing of initial injury management depend heavily on effective communication, collaboration, and the values placed on nutrition within sports settings. Findings highlight the urgent need for practical, evidence-based nutritional guidelines tailored to the unique demands and contexts of Irish sport.

Early injury identification, communication, consistent screening, timely nutritional assessment, and targeted interventions are essential to support tissue healing, recovery, and RTP. Structural factors such as sport type, gender, and organizational resources influence the consistency and effectiveness of support, highlighting the importance of PDs/PNs professional autonomy and practitioners’ role clarity.

Practitioner-informed strategies grounded in lived experience provide a practical, context-sensitive bridge between theory and real-world practice, enhancing athlete health and guiding the development of athlete-centered and adaptable tools.

In conclusion, this study underscores the importance of comprehensive screening, targeted nutrition, and consideration of psychological factors influencing recovery. Integrating these elements will help reduce disparities in injury management. At the policy, national bodies must ensure fair access to PD/PN services, allocate funding for nutrition roles, and embed nutrition support across all levels of Irish sport. These key actions will enable PDs and PNs to better support injured athletes and promote optimal health and performance outcomes post injury.

## Statement of significance

This is the first study to examine how performance dietitians and nutritionists in Ireland approach, assess, and manage athlete nutrition during the initial stages of injury. It offers practice-based insights for practitioners, working in injury-prone sporting environments and highlights the need for standardised, context-specific strategies to optimise recovery outcomes.

## Informed consent statement

Informed consent was obtained from all subjects involved in the study. Each participant completed informed consent forms prior to attending virtual interviews on Microsoft Teams.

## Author contributions

The authors’ responsibilities were as follows – EF: contributed to data acquisition, analysis, and manuscript writing; LR, ED: contributed to data analysis and manuscript writing; and all authors: reviewed and edited previous drafts of the manuscript, participated in discussions, provided feedback, and approved the manuscript before submission, involved in conceptualizing and designing the study, and read and agreed to the published version of the manuscript.

## Data availability

The original data, unfortunately, are unavailable to protect participant confidentiality. However, the data presented in the study article are included in the Supplementary Materials. Further inquiries can be directed to the corresponding author.

## Funding

The authors reported no funding received for this study.

## Conflict of interest

The authors report no conflicts of interest.
