# Supplementary material for: Contrasting Realities in Injury Management: Strategies Employed by Performance Nutritionists and Dietitians in Ireland (Part A)
Source: Curr Dev Nutr. 2025 Jul 24;9(9):107507. doi: 10.1016/j.cdnut.2025.107507 (PMC12414903; doi:10.1016/j.cdnut.2025.107507)
Supplement: Multimedia component 1 [file mmc1.docx]

| **Supplementary Table 1**  **Participant support roles:** PDs and PNs athlete/client types, settings, and employment contexts. | | | | | |
| --- | --- | --- | --- | --- | --- |
| **Role Type** | **PN ID** | **Athlete Type and Setting** | **Organisation**(s) | **Employment Context** |  |
| Full-time PNs in elite/ professional team sport | PN 2 | Rugby (male academy and senior), Gaelic football (male county) | IRFU, GAA | Full-time contract |  |
|  | PN 5 | Gaelic football (male county); individual recreational endurance, and rugby athletes | GAA | Full-time contract |  |
|  | PN 8 | Rugby (male academy, youth, senior); past: women’s soccer, senior ladies Gaelic football (intercounty) | IRFU, LGFA, GAA, Women’s FC | Full-time contract |  |
|  | PN 9 | Rugby (women’s XV and 7s, men’s 7s, and academy) | IRFU, GAA | Full-time contract |  |
|  | PN 13 | Women’s hockey, gymnastics (junior), endurance cyclists | Sport Ireland | Full-time contract |  |
|  | PN 14 | Rugby (male senior), Gaelic football (male senior), Olympic boxers, rowers, judo | Sport Ireland | Full-time contract |  |
| Part-time PNs in elite/ high-level team sport | PN 1 | Gaelic football (county senior and U20 male) | GAA | Part-time contract |  |
|  | PN 3 | Rugby (academy and senior males, women’s XV’s), athletics, Gaelic football and hurling (senior male) | IRFU, GAA, LGFA, Sport Ireland, Uni | Part-time contract |  |
|  | PN 4 | Gaelic football (senior males and females), Hurling (male) MMA, soccer (non-elite) | Women’s FC, IRFU, | Part-time contract |  |
|  | PN 7 | Hurlers (U20’s male), and senior Gaelic footballers (male), past individual soccer athletes | GAA | Part-time contract |  |
|  | PN 11 | Women’s soccer (senior), hockey (senior), rugby union teams; individual 1-1 athletes | Women’s FC, IRFU, private practice | Part-time contract |  |
|  | PN 12 | Gaelic footballers and hurlers (county male U20s and senior) | GAA | Part-time contract |  |
|  | PN 15 | Gaelic footballers and hurlers (senior and U20’s male), rugby union (male academy), individual Gaelic athletes and jockeys | IRFU, GAA | Part-time contract |  |
|  | PN 17 | Rugby (male academy and NTS), Gaelic football (female and male intercounty), Camogie (female), endurance athletes | IRFU | Part-time contract |  |
| Mixed individuals (self-employed full-time) | PN 6 | Endurance athletes (retired professionals and high level and amateur), kickboxing, HIFT, powerlifting athletes | N/A | Full-time self-employed |  |
|  | PN 10 | Combat athletes (professional and amateur), Gaelic footballers (county and club) | N/A | Full-time self-employed |  |
|  | PN 16 | Rugby (male, high-level, senior club), Gaelic football (male and female), hurling (male), camogie (female); weight class sports, Olympic athletes, power/ weightlifting, endurance, HIFT, multi-sport athletes | N/A | Full-time self-employed |  |
| **Abbreviations**: IRFU: Irish Rugby Football Union; FC: Football Club; GAA: Gaelic Athletic Association, MMA: Mixed Martial Arts, HIFT: high-intensity functional training. | | | | |  |

| 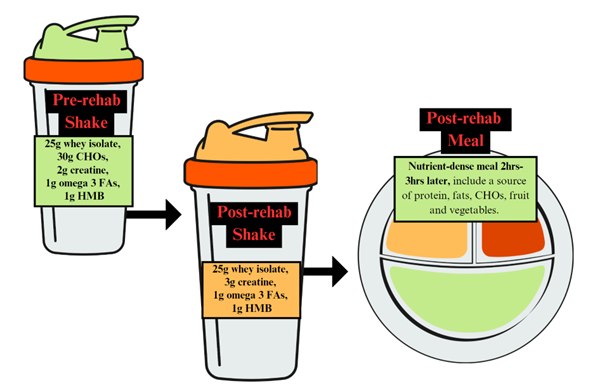 |
| --- |

**Supplementary Figure 1. Page 35.**

**Supplementary Material Legend**

**Supplementary Table 1:** Participant support roles: PDs and PNs athlete/client types, settings, and employment contexts (cited within text on page 13).

**Abbreviations**: IRFU: Irish Rugby Football Union; FC: Football Club; GAA: Gaelic Athletic Association; MMA: Mixed Martial Arts; HIFT: high-intensity functional training.

**Supplementary Figure 1:** Stage 3 protocol example cited by one participant [PN 6] (cited page 35).
